# Fermentation Profiles, Bacterial Community Compositions, and Their Predicted Functional Characteristics of Grass Silage in Response to Epiphytic Microbiota on Legume Forages

**DOI:** 10.3389/fmicb.2022.830888

**Published:** 2022-02-08

**Authors:** Siran Wang, Tao Shao, Junfeng Li, Jie Zhao, Zhihao Dong

**Affiliations:** Institute of Ensiling and Processing of Grass, College of Agro-Grassland Science, Nanjing Agricultural University, Nanjing, China

**Keywords:** silage, fermentative profiles, bacterial community compositions, predicted functional characteristics, Italian ryegrass

## Abstract

This study aimed to investigate the effect of epiphytic microbiota from alfalfa and red clover on the fermentative products, bacterial community compositions, and their predicted functional characteristics in Italian ryegrass silage. By microbiota transplantation and γ-ray irradiation sterilization, the irradiated Italian ryegrass was treated as follows: (1) sterile distilled water (STIR); (2) epiphytic microbiota on Italian ryegrass (IRIR); (3) epiphytic microbiota on alfalfa (IRAL); and (4) epiphytic microbiota on red clover (IRRC). The irradiated Italian ryegrass was ensiled for 1, 3, 7, 15, 30, and 60 days. STIR had similar chemical components with fresh Italian ryegrass. IRAL had higher lactic acid concentrations [64.0 g/kg dry matter (DM)] than IRIR (22.3 g/kg DM) and IRRC (49.4 g/kg DM) on day 3. IRRC had the lowest lactic acid concentrations (59.7 g/kg DM) and the highest pH (4.64), acetic acid (60.4 g/kg DM), ethanol (20.4 g/kg DM), and ammonia nitrogen (82.6 g/kg DM) concentrations and Enterobacteriaceae [9.51 log_10_ cfu/g fresh weight (FW)] populations among treatments on day 60. On days 3 and 60, *Lactobacillus* was dominant in both IRIR (42.2%; 72.7%) and IRAL (29.7%; 91.6%), while *Hafnia-Obesumbacterium* was predominant in IRRC (85.2%; 48.9%). IRIR and IRAL had lower abundances of “Membrane transport” than IRRC on day 3. IRIR and IRAL had lower abundances of phosphotransacetylase and putative ATP-binding cassette transporter and higher abundances of arginine deiminase on day 3. IRAL had the highest abundance of fructokinase on day 3. Overall, inoculating epiphytic microbiota from different legume forages changed the fermentative products, bacterial community compositions, and their predicted functional characteristics in Italian ryegrass silage. The microbial factors that result in the differences in fermentative profiles between legume forage and grass were revealed. Knowledge regarding the effect of epiphytic microbiota could provide more insights into the improvement of silage quality.

## Introduction

Ensiling of forages is extensively conducted worldwide to provide continuous feeds for ruminants. The ensiling process includes the quick establishment of anaerobic fermentation and anaerobic conditions of forages whereby lactic acid bacteria (LAB) metabolize water-soluble carbohydrates (WSCs) to various organic acids, primarily lactic acid, leading to silage acidification (Lin et al., 2021). The production of lactic acid in the ensiling process reduces the pH value that ensures silage preservation for a long period.

To date, legume forage and grass account for a large proportion of silage production. Legume forages, like alfalfa (*Medicago sativa* L.) and red clover (*Trifolium pratense* L.), have been widely cultivated due to their high crude protein (CP) contents. Regarding grass, they have abundant fermentable substrates and high palatability when fed as silages. It is acknowledged that the ensiling characteristics of legume forages are different from those of grasses. Numerous studies described that acetic acid is the primary fermentative product in legume forage silage, while lactic acid is produced in large quantities in most grass silages (Buxton et al., 2003). This difference is highly correlated with the chemical and microbial properties of raw materials. As for chemical components, legume forages usually had lower contents of WSC and dry matter (DM) associated with higher buffering capacity. Conversely, grasses possess sufficient WSC contents and optimal DM levels (McDonald et al., 1991). As for microbial compositions, Duniere et al. (2017) found that the epiphytic bacteria on different forages varied greatly. Nevertheless, there is limited information investigating their effects on fermentative products of legume forage and grass.

Recently, some studies have assessed the effects of exogenous microbiota on ensiling characteristics. The γ-ray irradiation sterilization technology was utilized to separate the substrate and epiphytic microbes in a variety of forages, and the microbiota transplantation method was applied to explore the single contribution of exogenous epiphytic microbiota to fermentative profiles and bacterial community dynamics (Nazar et al., 2021; Wang et al., 2022). However, Nazar et al. (2021) just evaluated the effects of epiphytic microbiota from tropical C_4_ forage crops (forage sorghum, napiergrass, whole-crop maize, and Sudan grass) on silage quality. Wang et al. (2022) focused on studying the impact of epiphytic microbiota from grasses (Italian ryegrass and oat) on the fermentative products of legume forage (red clover). Therefore, whether the epiphytic microbiota from legume forages could fit well and reconstitute in grass silages is unclear. We assumed that transplanting the epiphytic microbiota from legume forages into grass silages could reconstitute a bacterial community with similar functions as found in legume forage silages.

In temperate areas, Italian ryegrass (*Lolium multiflorum* Lam.) is the principal grass during silage making. Alfalfa and red clover can typically represent the legume forages. Hence, this study aimed to evaluate the effects of epiphytic bacteria from different legume forages on fermentation profiles, bacterial community dynamics, and predicted functional characteristics in grass silage. Knowledge regarding the effects of epiphytic microbiota could provide more insights into understanding the microbial factors that cause the difference between legume forage and grass silages. Concretely, the epiphytic microbiota on alfalfa and red clover were collected and added to the irradiated Italian ryegrass, and their influences on fermentative products, bacterial community compositions, and predicted metabolic pathways were recorded.

## Materials and Methods

### Inoculum Preparation and Silage Making

Italian ryegrass, alfalfa, and red clover were cultivated in the experimental field (Baima Teaching and Research Base) of Nanjing Agricultural University. This area has a subtropical monsoon climate with an average temperature of 15.7°C, mean annual precipitation of 1,105 mm, and an average elevation of 24.8 m. Fresh Italian ryegrass (IR) was harvested at the heading stage, while alfalfa (AL) and red clover (RC) were harvested at the early bloom stage of maturity. Without wilting, each of the three forages was separately chopped (length: 1 ∼ 2 cm) by a chaff cutter, and a homogeneous mixture was made for preparing inoculum and making silage.

The inoculum of Italian ryegrass, alfalfa, and red clover was prepared according to the method of Mogodiniyai Kasmaei et al. (2016), with small modifications. Specifically, 900 ml of Ringer solution added by Tween-80 (concentration: 0.5 ml/L) was mixed with 111 g of fresh forage. Considering the microbial loss, the nearly 100% epiphytic microbiota on 100 g of fresh material could be represented by the eluted liquid from 111 g of fresh material theoretically. It was calculated based on two important assumptions in the previous studies (Mogodiniyai Kasmaei et al., 2016; Wang et al., 2022): (1) epiphytic microorganisms were completely eluted from raw material and uniformly distributed in the eluent; and (2) after centrifugation, the microbial recovery was just 90%. Subsequently, the 111-g samples mixed with Ringer solutions were put in the shaker (rate, 120 rpm; time, 1.5 h) and centrifuged at 12,000 × *g* for 10 min. After centrifugation, the supernatant in tubes was abandoned, and the residues were combined and dissolved in 3 ml of Ringer solution. Thus, this 3-ml inoculum collected from 111 g of fresh forage represented the whole epiphytic microbiota on 100 g of fresh material.

After being chopped, fresh Italian ryegrass (100 g) was packed into a vacuum-packed bag (30 cm × 32 cm) and sealed with a vacuum sealer. Then, 72 vacuum-packed bags (4 treatments × 3 replicates × 6 ensiling time = 72) were immediately transported to the irradiation company (Nanjing Xiyue Irradiation Technology Co., Ltd., Nanjing, China) by car within 2 h. The samples were sterilized by using a ^60^Co source (dose, 32 kGy; time, 15 min) according to the description of Junges et al. (2017). To equally evaluate the effects of different epiphytic microbiota on the same substrate, our experiment was conducted based on the equivalent principle that “100 g raw material should be inoculated by the whole epiphytic microbiota from the 100 g fresh material.” Hence, 100 g of sterilized Italian ryegrass herein was inoculated by the prepared 3-ml inoculum from Italian ryegrass, alfalfa, and red clover. As the substrate, 100 g of irradiated Italian ryegrass was inoculated by the following: (1) sterile distilled water (STIR); (2) epiphytic microbiota on Italian ryegrass (IRIR); (3) epiphytic microbiota on alfalfa (IRAL); and (4) epiphytic microbiota on red clover (IRRC). The prepared 3-ml exogenous microbiota or sterile distilled water was added to the irradiated Italian ryegrass. The abovementioned operations were accomplished in the pretreated clean bench, which was irradiated by ultraviolet light for 2 h and cleaned by ethanol to avoid contamination. After inoculation, the vacuum-packed bags were evacuated and sealed. During the inoculating, vacuuming, and sealing, the microbes in the air of the lab were negligible. Finally, the sealed samples were conserved at room temperature (∼24°C) and randomly opened after 1, 3, 7, 15, 30, and 60 days of fermentation.

### Fermentation Quality Analyses

When opening the bags, each sample was mixed thoroughly by hand with sterile gloves in the clean plastic container. The plastic container was irradiated by ultraviolet light for 1 h in the clean bench in advance. Firstly, a 25-g sample was blended with 75 ml of distilled water and preserved at 4°C for 6 h. Then, the extracts were filtered by one filter paper and two layers of cheesecloth. The filtrates were finally stored in 50-ml centrifuge tubes at −20°C for the following analyses. The pH of the sample was determined by a glass electrode pH meter (PHSJ-5; LEICI, Shanghai, China). The buffering capacity of fresh forage was determined according to the method of Playne and McDonald (1966). The organic acid and ethanol contents were determined with the Agilent HPLC 1260 (Agilent Technologies, Inc., Santa Clara, CA, United States; column, Carbomix H-NP5, Sepax Technologies, Inc., Santa Clara, CA, United States; detector, refractive index detector, Agilent Technologies, Inc., Santa Clara, CA, United States; eluent, 2.5 mmol/L H_2_SO_4_, 0.5 ml/min; temperature, 55°C). The NH_3_–N contents were determined according to the description of Broderick and Kang (1980).

Secondly, a part of fresh forage or silage was analyzed immediately for the DM content in a forced-draft oven to a constant weight drying at 60°C for at least 48 h and then ground to pass a 1-mm screen in a laboratory knife mill (FW100, Taisite Instrument Co., Ltd., Tianjin, China) for later analysis. The milled sample was used for total nitrogen (TN), WSC, and fiber analysis. The TN concentrations were determined according to the Kjeldahl method (Krishnamoorthy et al., 1982). The CP contents were calculated by multiplying the TN value by 6.25. The WSC contents were determined using the anthrone colorimetry method (Thomas, 1977). The acid detergent fiber (ADF), acid detergent lignin (ADL), and neutral detergent fiber (NDF) contents were determined by the method of Van Soest et al. (1991).

Thirdly, to evaluate the microbial populations, a 10-g sample was blended with 90 ml of sterile saline and shaken for 1 h at 120 rpm. After shaking, 1 ml of the solution was taken from each bottle and used for counting microorganisms. The residual liquid was filtered by sterile gauze and used for DNA extraction. Yeast, LAB, aerobic bacteria, and Enterobacteriaceae colonies were cultured and counted by the methods of Wang et al. (2020a).

### Bacterial Community Analysis

The bacterial community varies greatly at the initial stage of fermentation, and the final state of silage is important for researchers to evaluate the fermentation quality. Hence, the raw materials [fresh material of Italian ryegrass (IRFM), fresh material of alfalfa (ALFM), and fresh material of red clover (RCFM)], and silage samples on day 3 (IRIR-3, IRAL-3, and IRRC-3) and day 60 (IRIR-60, IRAL-60, and IRRC-60) were selected to investigate their bacterial diversity and functional characteristics through high-throughput sequencing. The details in determining the bacterial diversity of these samples were according to the description of Wang et al. (2020a). In brief, the preserved liquid for extracting DNA was centrifuged (rate, 12,000 × *g*; time, 10 min), and the pellet was collected and used to extract DNA by kit (MP Biomedicals, Santa Ana, CA, United States) based on the description of manufacturer’s protocols. The 338F and 806R were selected as primers to amplify the V_3_–V_4_ regions of bacterial 16S ribosomal RNA. DNA samples were paired-end sequenced on the platform of Illumina (San Diego, CA, United States) MiSeq PE300.

The FLASH software was applied to check the raw reads, and the quality sequences (scores > 80) were saved based on the QIIME quality control process. The operational taxonomic units (OTUs) with 97% similarity were clustered by the UPARSE pipeline software. Then, the UCHIME software was used to identify and remove the chimeric sequences. The alpha diversities including rarefaction curves, Shannon curves, OTUs, Shannon, Chao1, Sobs, Simpson, Ace, Coverage, and Shannoneven indices were performed using the Mothur software. The bacterial community compositions were determined at the genus and phylum levels through the Silva 138 database (confidence, >70%). The community heatmap and hierarchical cluster analysis on the genus level (the most abundant 30 genera), principal coordinate analysis (PCoA), Spearman’s correlation heatmap and hierarchical cluster analysis, and redundancy analysis (RDA) were graphically displayed by the R software (version 4.0.5) according to the packages. The metabolic potential of bacterial community and the composition of functional genes were postulated by assigning 16S rRNA marker gene sequences to functional annotations of sequenced metagenomic sequences based on the Kyoto Encyclopedia of Genes and Genomes (KEGG) pathways Orthology (KO) classification, using Tax4Fun (version 0.3.1) as described by Aßhauer et al. (2015). The comparisons in the KEGG pathways and enzymes were graphically presented via Graphpad Prism (version 8, IBM, Armonk, NY, United States). The sequence data were uploaded in the Sequence Read Archive (SRA) under the accession number PRJNA778663.

### Statistical Analysis

The Statistical Packages for the Social Sciences (SPSS, version 19.0, SPSS Inc., Chicago, IL, United States) was used to examine the differences among treatments. The comparison between fresh and sterile Italian ryegrass was conducted by one-way ANOVA. The data on fermentation products, chemical compositions, and microbial populations were subjected to two-way ANOVA. The data on bacterial communities, relative abundances of the KEGG pathway, and key enzymes were subjected to one-way ANOVA. The microbial populations were acquired as colony-forming units (cfu) based on fresh weight (FW). The method of Tukey’s multiple comparisons was applied to analyze the statistical difference. Differences were considered as significant at *p* < 0.05.

## Results

### Chemical Components and Microbial Populations of Fresh Forages and Sterile Italian Ryegrass

As seen in Table 1, fresh red clover had the relatively lowest LAB population (4.64 log_10_ cfu/g FW) among three fresh materials. The counts of aerobic bacteria, yeasts, and Enterobacteriaceae in fresh alfalfa red clover were higher than 6.50 log_10_ cfu/g FW. The WSC content of fresh Italian ryegrass was 122 g/kg DM, epiphytic LAB count on fresh Italian ryegrass was 5.22 log_10_ cfu/g FW, and undesirable microbes containing Enterobacteriaceae, yeasts, and aerobic bacteria were higher than 6.40 log_10_ cfu/g FW. Besides, the chemical compositions between sterile and fresh Italian ryegrass were similar (*p* > 0.05).

**TABLE 1:** Chemical and microbial compositions of fresh forages and sterile Italian ryegrass before ensiling.

Items	Fresh alfalfa	Fresh red clover	Fresh Italian ryegrass	Sterile Italian ryegrass	*p*-value
					IRFM vs. STIR
pH	6.21	6.02	6.07	6.06	0.058
Dry matter (g/kg FW)	264	262	247	244	0.862
Water-soluble carbohydrates (g/kg DM)	79.5	73.3	122	120	0.541
Buffering capacity (mEq/kg DM)	314	311	83.8	82.4	0.214
Neutral detergent fiber (g/kg DM)	398	396	561	564	0.856
Acid detergent fiber (g/kg DM)	227	242	344	341	0.867
Acid detergent lignin (g/kg DM)	78.5	84.1	87.3	86.5	0.217
Crude protein (g/kg DM)	224	247	90.0	87.0	0.334
Lactic acid bacteria (log_10_ cfu/g FW)	6.51	4.64	5.22	ND	–
Aerobic bacteria (log_10_ cfu/g FW)	7.66	7.51	7.14	ND	–
Yeasts (log_10_ cfu/g FW)	6.57	6.54	6.41	ND	–
Enterobacteriaceae (log_10_ cfu/g FW)	7.79	6.72	8.25	ND	–

*DM, dry matter; FW, fresh weight; mEq, milligram equivalent; cfu, colony-forming units; ND, not detected; IRFM, fresh Italian ryegrass; STIR, sterile Italian ryegrass.*

### The Alpha Diversities of Fresh Materials and Silages

**FIGURE 1:**
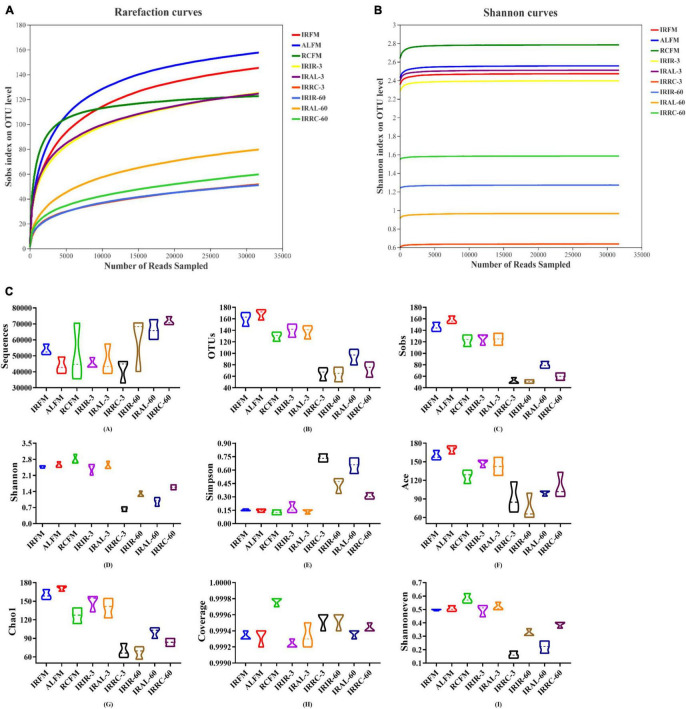
Richness and diversity indices of bacterial communities in fresh materials and Italian ryegrass silages during ensiling. **(A)** Rarefaction curves. **(B)** Shannon curves. **(C)** Richness and diversity indices of bacterial communities. IRFM, fresh material of Italian ryegrass; ALFM, fresh material of alfalfa; RCFM, fresh material of red clover; IRIR, sterile Italian ryegrass inoculated by epiphytic bacteria from Italian ryegrass; IRAL, sterile Italian ryegrass inoculated by epiphytic bacteria from alfalfa; IRRC, sterile Italian ryegrass inoculated by epiphytic bacteria from red clover; 3, 3 days of ensiling; 60, 60 days of ensiling.

As shown in Figures 1A,B, with the number of reads sampled increasing, the rarefaction curves in all samples exhibited an upward trend during the initial stage of detection and then remained stable at the end. The Shannon curves reached stable levels at the early stage of detection. As described in Figure 1C, fresh materials had similar alpha diversities with silage samples on day 3 (except the IRRC-3 group), but notably higher alpha diversities (mainly including OTUs, Sobs, Shannon, Ace, Chao1, and Shannoneven) than silage samples on day 60. In particular, the IRRC group had higher Simpson and lower Shannon and Shannoneven indices than the IRIR and IRAL groups on day 3. The Coverage values for all samples were higher than 99.91%.

### The Bacterial Community Compositions in the Fresh Materials and Silages

**FIGURE 2:**
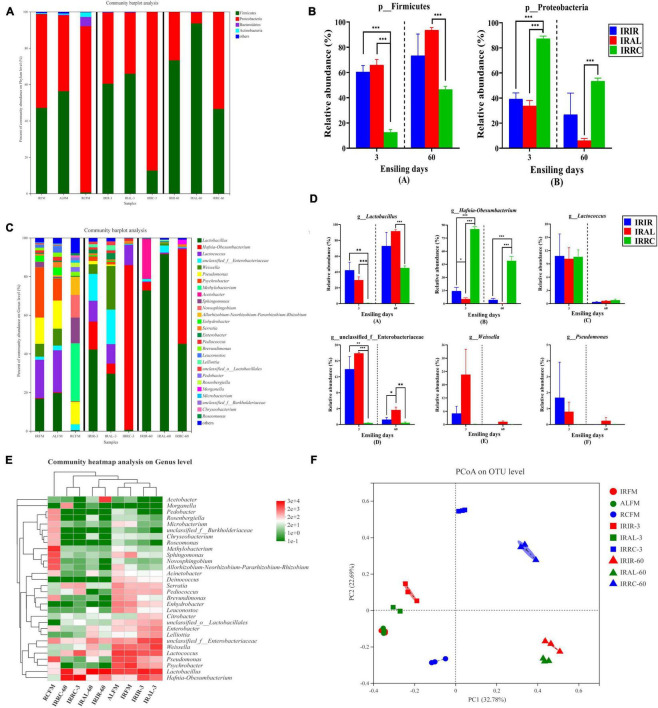
Phylum and genus level compositions **(A–D)** of the bacterial community in Italian ryegrass silages. **(E)** Community heatmap analysis on genus level. **(F)** Principal coordinate analysis (PCoA) plot on operational taxonomic unit (OTU) level. IRFM, fresh material of Italian ryegrass; ALFM, fresh material of alfalfa; RCFM, fresh material of red clover; IRIR, sterile Italian ryegrass inoculated by epiphytic bacteria from Italian ryegrass; IRAL, sterile Italian ryegrass inoculated by epiphytic bacteria from alfalfa; IRRC, sterile Italian ryegrass inoculated by epiphytic bacteria from red clover; 3, 3 days of ensiling; 60, 60 days of ensiling. *, 0.01 < *p* < 0.05; ^**^, 0.001 < *p* < 0.01; ^***^, *p* < 0.001.

The bacterial community on the phylum level in raw materials and silage samples is described in Figures 2A,B. Proteobacteria and Firmicutes were both the predominant phyla in IRFM (51.6%; 47.1%) and ALFM (41.8%; 56.3%) groups, and they totally dominated >98.0% proportions of the entire epiphytic bacterial community. Proteobacteria accounted for 91.4% of the bacterial community and was the only dominant phylum in the RCFM group. After the ensiling process, the relative abundances of Firmicutes phylum in the treated groups on days 3 and 60 increased in varying degrees.

The bacterial community on the genus level in raw materials and silage samples is described in Figures 2C,D. Higher than 11.5% of *Pseudomonas* were found in all fresh materials. The predominant genus in RCFM was *Methylobacterium* (29.9%). About 13.4% of *Sphingomonas* was found in RCFM. After the ensiling process, the relative abundances of *Lactobacillus* in the IRIR and IRAL groups were significantly (*p* < 0.05) or numerically (*p* > 0.05) higher than those in the IRRC group on days 3 and 60. The relative abundance of *Hafnia-Obesumbacterium* in the IRRC group was significantly (*p* < 0.001) higher than that in the IRIR and IRAL groups during the fermentation. There was no significant (*p* > 0.05) difference in the relative abundances of *Lactococcus* among the three treated groups on day 3. The relative abundances of Enterobacteriaceae in the IRIR and IRAL groups were significantly (*p* < 0.05) higher than those in the IRRC group on day 3. The relative abundance of *Weissella* in the IRAL group was numerically (*p* > 0.05) higher than that in the IRIR and IRRC groups on day 3.

According to the results of the community heatmap and hierarchical cluster analysis (Figure 2E), the bacterial community in the RCFM group was distinctly different from that in the IRFM and ALFM groups. As shown in Figure 2F, the variance of the bacterial community was exhibited by the PCoA plot on the OTU level. Components 1 and 2 explained 32.78 and 22.69% of the total variance, respectively. All the silage samples were well separated from the raw materials and divided into different quadrants in the PCoA plot.

### Fermentation Characteristics and Their Correlations With Bacterial Community Compositions

The dynamic changes of fermentation products in Italian ryegrass silages are described in Table 2. The STIR group remained in an unfermented state and had similar chemical compositions with fresh Italian ryegrass during the entire ensiling process. During the initial 3 days of fermentation, lactic acid contents accumulated rapidly, and pH values declined dramatically in the three treated groups. In the IRAL and IRRC groups, the lactic acid concentrations were reduced on day 30 of ensiling. The IRAL group had significantly (*p* < 0.05) higher lactic acid contents than the IRIR and IRRC groups during the initial stage. After 60 days of fermentation, the IRIR and IRAL groups had notably (*p* < 0.05) higher lactic acid contents and lower pH values than the IRRC group. During the entire ensiling process, the contents of acetic acid in the three treated groups gradually increased and reached higher than 60.4 g/kg DM on day 60. The acetic acid contents in the IRIR and IRAL groups were significantly (*p* < 0.05) lower than those in the IRRC group. Trace and acceptable amounts of propionic acid (<4.00 g/kg DM) and butyric acid (<2.00 g/kg DM) were detected in all fermented groups (data not shown). Ethanol contents in all treated silages were lower than 21.0 g/kg DM. IRRC had evidently (*p* < 0.05) higher ethanol concentrations than IRAL and IRIR on day 60.

**TABLE 2:** Effects of exogenous microbiota on pH, organic acid, and ethanol contents in Italian ryegrass silages.

Items	Treatments	Ensiling days						*SEM*	*p*-value		
		1	3	7	15	30	60		*T*	*D*	*T* × *D*
pH	STIR	6.15^Aa^	6.09^ABa^	6.05^BCa^	6.00^Ca^	6.14^Aa^	6.13^Aa^	0.001	<0.001	<0.001	<0.001
	IRIR	6.01^Ab^	4.42^Bc^	4.06^Dd^	4.14^Cc^	4.11^CDc^	4.09^CDc^				
	IRAL	4.84^Ac^	4.33^Bd^	4.23^Cc^	4.08^Dc^	4.01^Ed^	4.04^DEc^				
	IRRC	6.00^Ab^	4.52^Cb^	4.47^Cb^	4.26^Db^	4.67^Bb^	4.64^Bb^				
Lactic acid (g/kg DM)	STIR	1.58^ABb^	1.26^ABd^	2.41^Ad^	2.31^Ad^	1.06^Bd^	1.69^ABd^	2.768	<0.001	<0.001	<0.001
	IRIR	2.65^Eb^	22.3^Dc^	42.9^Cc^	72.3^Bb^	118^Ab^	120^Aa^				
	IRAL	52.1^Da^	64.0^Ca^	101^Ba^	105^Ba^	122^Aa^	99.8^Bb^				
	IRRC	2.61^Eb^	49.4^Db^	64.7^ABb^	54.1^CDc^	69.0^Ac^	59.7^BCc^				
Acetic acid (g/kg DM)	STIR	2.59^ABc^	2.84^ABc^	3.60^ABc^	4.50^Ab^	1.50^Bc^	2.84^ABd^	1.237	<0.001	<0.001	<0.001
	IRIR	14.3^Da^	28.4^Ca^	11.6^Db^	14.3^Da^	35.8^Bb^	51.1^Ab^				
	IRAL	9.52^Db^	9.60^Db^	17.1^Ca^	14.3^Ca^	45.1^Aa^	39.9^Bc^				
	IRRC	11.3^Db^	10.8^Db^	16.5^Ca^	16.0^Ca^	45.1^Ba^	60.4^Aa^				
Lactic acid/acetic acid	STIR	0.78^b^	0.46^c^	0.74^c^	0.55^d^	0.79^d^	0.63^c^	0.155	<0.001	<0.001	<0.001
	IRIR	0.19^Db^	0.79^Dc^	3.74^Bb^	5.05^Ab^	3.33^Ba^	2.35^Ca^				
	IRAL	5.49^Ba^	6.72^ABa^	5.95^ABa^	7.42^Aa^	2.70^Cb^	2.51^Ca^				
	IRRC	0.23^Db^	4.60^Ab^	3.92^ABb^	3.40^Bc^	1.54^Cc^	0.99^CDb^				
Ethanol (g/kg DM)	STIR	5.83^ABb^	6.19^ABc^	6.78^Ac^	4.89^Bc^	5.31^Bc^	5.21^Bc^	1.367	<0.001	<0.001	<0.001
	IRIR	12.3^BCa^	10.4^BCb^	9.46^Cc^	9.48^Cb^	13.1^ABb^	15.1^Ab^				
	IRAL	7.79^Bb^	8.44^Bbc^	13.1^Ab^	11.5^Ab^	13.0^Ab^	13.9^Ab^				
	IRRC	12.8^Ca^	15.5^ABCa^	17.9^ABa^	14.8^BCa^	17.8^ABa^	20.4^Aa^				

*Means with different letters in the same row (A–E) or column (a–d) differ (p < 0.05).*

*DM, dry matter; STIR, sterile Italian ryegrass; IRIR, sterile Italian ryegrass inoculated by epiphytic microbiota from Italian ryegrass; IRAL, sterile Italian ryegrass inoculated by epiphytic microbiota from alfalfa; IRRC, sterile Italian ryegrass inoculated by epiphytic microbiota from red clover. T, microbiota; D, ensiling days; T × D, the interaction between microbiota and ensiling days.*

The changes of microbial populations and chemical components in Italian ryegrass silage are shown in Table 3. The DM contents in the STIR group remained stable as compared with the fresh Italian ryegrass. The DM contents of all fermented groups were dramatically (*p* < 0.05) reduced after ensiling. Compared with the IRIR and IRRC groups on day 3 or 60, notably (*p* < 0.05) higher DM contents were observed in the IRAL group. The NH_3_–N concentrations in the STIR group were gradually increased during ensiling. All the fermented silages had less than 90.0 g/kg of TN NH_3_–N concentrations. The significantly (*p* < 0.05) lower NH_3_–N concentrations were observed in the IRAL group than the IRIR and IRRC groups on day 3 or 60. The WSC levels in the STIR group remained steady during ensiling. IRAL had a notably (*p* < 0.05) higher WSC concentration than IRRC and IRIR after 3 days of ensiling. IRIR and IRAL had significantly (*p* < 0.05) higher LAB counts than the IRRC group. The significantly highest (*p* < 0.001) LAB counts were observed in the IRAL-3 group. The significantly (*p* < 0.05) or numerically (*p* > 0.05) higher Enterobacteriaceae and yeast populations were found in the IRRC group compared with the other treated groups on days 3 and 60.

**TABLE 3:** Effects of exogenous microbiota on chemical and microbial compositions in Italian ryegrass silages.

Items	Treatments	Ensiling days						*SEM*	*p*-value		
		1	3	7	15	30	60		*T*	*D*	*T* × *D*
Dry matter (g/kg FW)	STIR	242^a^	245^a^	244^a^	243^a^	243^a^	244^a^	2.181	0.001	<0.001	0.004
	IRIR	225^Ad^	227^Ac^	222^Bc^	223^Bb^	221^Bc^	220^Bc^				
	IRAL	230^Ac^	232^Ab^	229^Ab^	226^Bb^	227^Bb^	227^Bb^				
	IRRC	236^Ab^	224^Bd^	223^Bc^	224^Bb^	219^Cc^	214^Dd^				
Ammonia nitrogen (g/kg TN)	STIR	3.26^Ec^	5.34^Ec^	11.5^Dd^	15.7^Cd^	20.9^Bd^	27.5^Ad^	1.347	<0.001	<0.001	<0.001
	IRIR	12.6^Ea^	24.8^Da^	28.0^Db^	36.0^Cb^	44.8^Bb^	55.3^Ab^				
	IRAL	6.90^Eb^	14.7^Db^	19.7^CDc^	24.0^Cc^	36.4^Bc^	44.1^Ac^				
	IRRC	12.5^Fa^	23.4^Ea^	49.0^Da^	62.5^Ca^	73.4^Ba^	82.6^Aa^				
Water-soluble carbohydrates (g/kg DM)	STIR	120^a^	122^a^	122^a^	121^a^	123^a^	121^a^	2.613	<0.001	<0.001	<0.001
	IRIR	84.5^Ab^	37.7^Bc^	30.3^Cb^	24.5^Cb^	14.2^Db^	10.4^Db^				
	IRAL	65.2^Ac^	50.0^Bb^	32.3^Cb^	21.3^Db^	12.9^Eb^	9.55^Fb^				
	IRRC	71.9^Ac^	32.1^Bd^	20.1^Cc^	14.3^Dc^	11.5^DEb^	7.17^Ec^				
Lactic acid bacteria (log_10_ cfu/g FW)	STIR	ND	ND	ND	ND	ND	ND	0.029	<0.001	<0.001	<0.001
	IRIR	8.15^Bb^	8.15^Bb^	9.37^Aa^	9.40^Aa^	9.30^Ab^	9.90^Aa^				
	IRAL	8.52^Ba^	9.40^Aa^	9.25^Aa^	9.78^Aa^	9.61^Aa^	9.59^Aa^				
	IRRC	7.13^Dc^	7.13^Dc^	7.55^Cb^	7.18^CDb^	9.71^Aa^	8.34^Bb^				
Enterobacteriaceae (log_10_ cfu/g FW)	STIR	ND	ND	ND	ND	ND	ND	0.036	<0.001	<0.001	<0.001
	IRIR	8.58^Aa^	8.06^Ab^	6.62^Bb^	6.53^Bb^	4.33^Cc^	4.41^Cc^				
	IRAL	6.96^Ac^	6.63^Ac^	5.69^Bc^	4.69^Cc^	4.91^Cb^	4.56^Cb^				
	IRRC	8.06^Cb^	8.74^Ba^	7.46^Da^	8.46^BCa^	9.34^Aa^	9.51^Aa^				
Yeasts (log_10_ cfu/g FW)	STIR	ND	ND	ND	ND	ND	ND	0.057	<0.001	<0.001	<0.001
	IRIR	6.44^Aa^	5.43^Bb^	5.52^Ba^	4.31^Cb^	4.23^Cb^	4.05^Cab^				
	IRAL	5.66^Ab^	4.56^Cc^	4.79^BCb^	5.37^ABa^	4.31^Cb^	3.49^Db^				
	IRRC	6.55^Aa^	6.52^Aa^	4.67^Cb^	4.93^BCa^	5.63^Ba^	4.48^Ca^				

*Means with different letters in the same row (A–F) or column (a–d) differ (p < 0.05).*

*DM, dry matter; FW, fresh weight; TN, total nitrogen; cfu, colony-forming units; ND, not detected; STIR, sterile Italian ryegrass; IRIR, sterile Italian ryegrass inoculated by epiphytic microbiota from Italian ryegrass; IRAL, sterile Italian ryegrass inoculated by epiphytic microbiota from alfalfa; IRRC, sterile Italian ryegrass inoculated by epiphytic microbiota from red clover. T, microbiota; D, ensiling days; T × D, the interaction between microbiota and ensiling days.*

**FIGURE 3:**
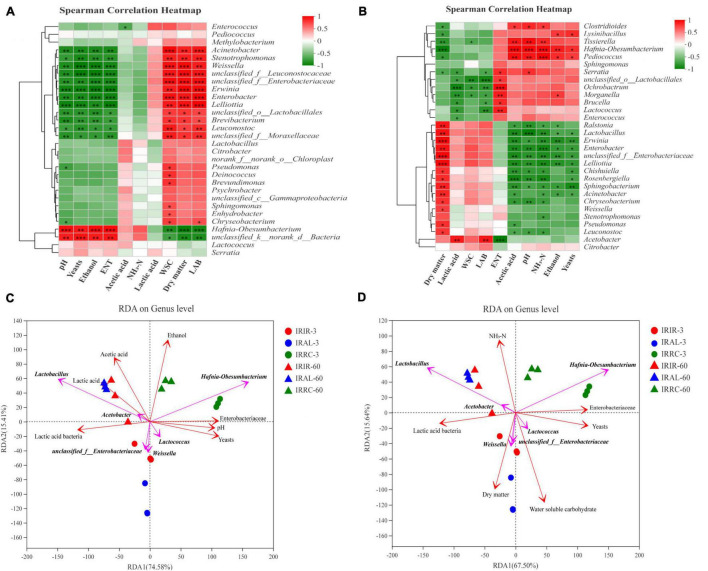
Spearman’s correlation heatmap in Italian ryegrass silages on day 3 **(A)** and 60 **(B)**, respectively. **(C,D)** Redundancy analysis (RDA) on genus level in Italian ryegrass silages. IRIR, sterile Italian ryegrass inoculated by epiphytic bacteria from Italian ryegrass; IRAL, sterile Italian ryegrass inoculated by epiphytic bacteria from alfalfa; IRRC, sterile Italian ryegrass inoculated by epiphytic bacteria from red clover; 3, 3 days of ensiling; 60, 60 days of ensiling. *, 0.01 < *p* < 0.05; ^**^, 0.001 < *p* < 0.01; ^***^, *p* < 0.001.

The relationships between the bacterial community and fermentative products in Italian ryegrass silages on days 3 and 60 are described in Figures 3A,B, respectively. After 3 days of ensiling, a negative correlation (*p* < 0.01) was noticed between the abundance of *Weissella* and pH values. On day 60, the abundance of *Hafnia-Obesumbacterium* had a positive correlation (*p* < 0.01) with acetic acid, NH_3_–N, and ethanol contents. A negative relationship (*p* < 0.001) was observed between the abundance of *Lactobacillus* and pH values on day 60.

The results of RDA are shown in Figures 3C,D. The arrow direction of *Hafnia-Obesumbacterium* showed an acute angle correlation with arrow directions of ethanol and pH values. An obtuse angle relationship was observed between *Hafnia-Obesumbacterium* and DM arrow directions. An acute angle relationship was found between *Hafnia-Obesumbacterium* and NH_3_–N arrow directions.

### Kyoto Encyclopedia of Genes and Genomes Metabolic Pathways of Bacterial Community in the Raw Materials and Silages

**FIGURE 4:**
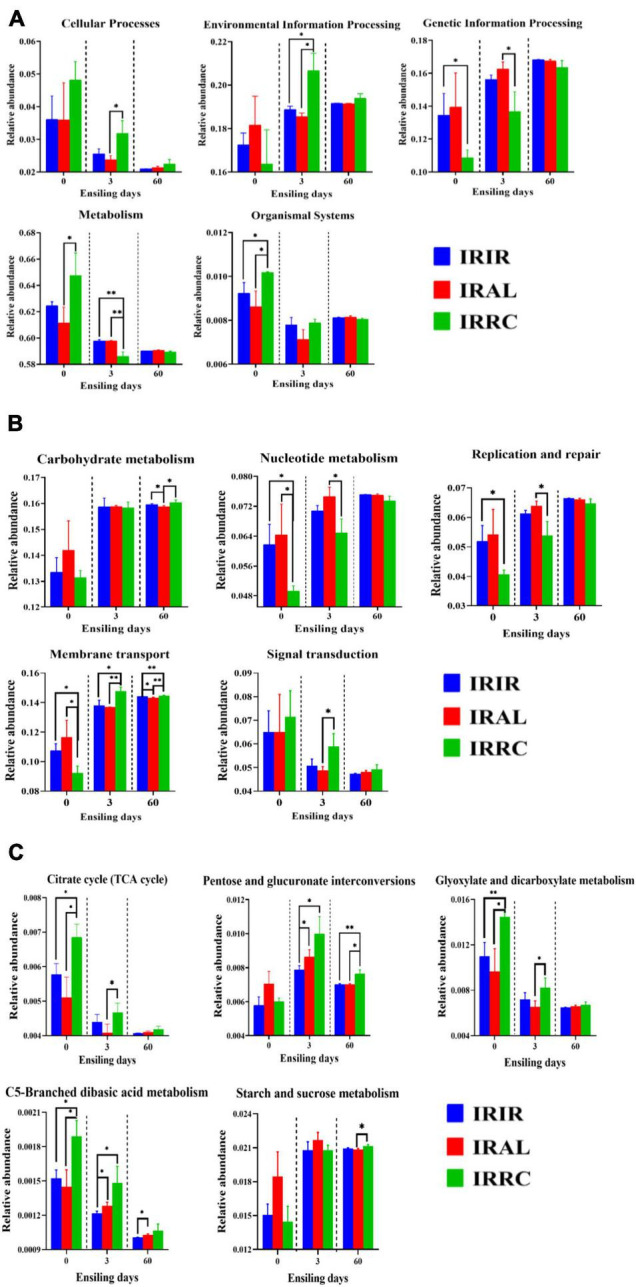
The changes of KEGG metabolic pathways on the first **(A)**, second **(B)**, and carbohydrate pathway **(C)** levels obtained with Tax4Fun in different groups. KEGG, Kyoto Encyclopedia of Genes and Genomes; IRIR, sterile Italian ryegrass inoculated by epiphytic bacteria from Italian ryegrass; IRAL, sterile Italian ryegrass inoculated by epiphytic bacteria from alfalfa; IRRC, sterile Italian ryegrass inoculated by epiphytic bacteria from red clover. *, 0.01 < *p* < 0.05; ^**^, 0.001 < *p* < 0.01.

The changes of predicted metabolic pathways on the first level are described in Figure 4A. Before the ensiling process, the pathway levels of epiphytic bacteria on three fresh materials varied greatly. After 3 days of ensiling, the IRIR and IRAL groups had significantly (*p* < 0.05) or numerically (*p* > 0.05) lower abundances of “Cellular Process” and “Environmental Information Processing” and higher abundances of “Genetic Information Processing” and “Metabolism” than the IRRC group. After 60 days of ensiling, a low variation of metabolic pathways on the first level is found among the different treatments.

The changes of metabolic pathways on the second pathway level in bacterial communities of different groups are described in Figure 4B. The “Carbohydrate metabolism” pathways in different treatments were promoted after ensiling. After 3 days of fermentation, IRAL had remarkably (*p* < 0.05) higher abundances of “Nucleotide metabolism” and “Replication and repair” than the IRRC group. The relative abundances of “Membrane transport” and “Signal transduction” in IRAL-3 were significantly (*p* < 0.05) less than those in IRRC-3. Compared with the IRIR and IRAL groups, a significantly (*p* < 0.05) or numerically (*p* > 0.05) higher abundance of “Signal transduction” was observed in the IRRC group on day 3.

As seen in Figure 4C, the carbohydrate metabolic pathways were specifically analyzed on the third pathway level. The relative abundances of the citrate cycle (TCA cycle) in all treatments were inhibited after ensiling as compared with fresh materials. The IRAL group had a significantly (*p* < 0.05) lower abundance of TCA pathway than the IRRC group on day 3. The IRRC group had evidently (*p* < 0.05) higher abundances of pentose and glucuronate interconversions, glyoxylate and dicarboxylate metabolism, and C5-branched dibasic acid metabolism than the IRAL group after 3 days of fermentation. There was no significant (*p* > 0.05) difference in starch and sucrose metabolism among the treatments on day 3.

**FIGURE 5:**
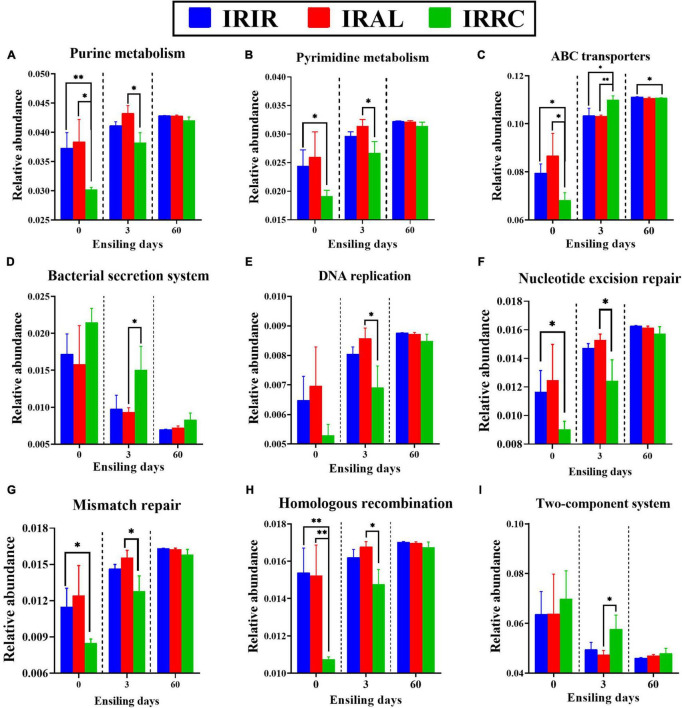
The changes of some KEGG metabolic pathways on the nucleotide metabolism **(A,B)**, membrane transport **(C,D)**, replication and repair **(E–H)**, and signal transduction **(I)** obtained with Tax4Fun in different groups. KEGG, Kyoto Encyclopedia of Genes and Genomes; IRIR, sterile Italian ryegrass inoculated by epiphytic bacteria from Italian ryegrass; IRAL, sterile Italian ryegrass inoculated by epiphytic bacteria from alfalfa; IRRC, sterile Italian ryegrass inoculated by epiphytic bacteria from red clover. *, 0.01 < *p* < 0.05; ^**^, 0.001 < *p* < 0.01.

As described in Figure 5, some metabolic pathways with notable differences on the second level were further analyzed on the third pathway level. The IRAL group had significantly (*p* < 0.05) higher abundances of purine metabolism and pyrimidine metabolism than the IRRC group on day 3. After the ensiling process, the relative abundances of ABC transporters in all treatments were promoted, while the relative abundances of the bacterial secretion system in all treatments were inhibited, especially on day 60. After 3 days, IRAL had significantly (*p* < 0.05) fewer ABC transporters and bacterial secretion systems than IRRC. The IRAL group had significantly (*p* < 0.05) higher abundances of DNA replication, nucleotide excision repair, mismatch repair, and homologous recombination than the IRRC group on day 3. The IRAL group had a significantly (*p* < 0.05) lower abundance of the two-component system than the IRRC group after 3 days of fermentation.

### The Activity of Key Enzymes of Bacterial Community in the Raw Materials and Silages

**FIGURE 6:**
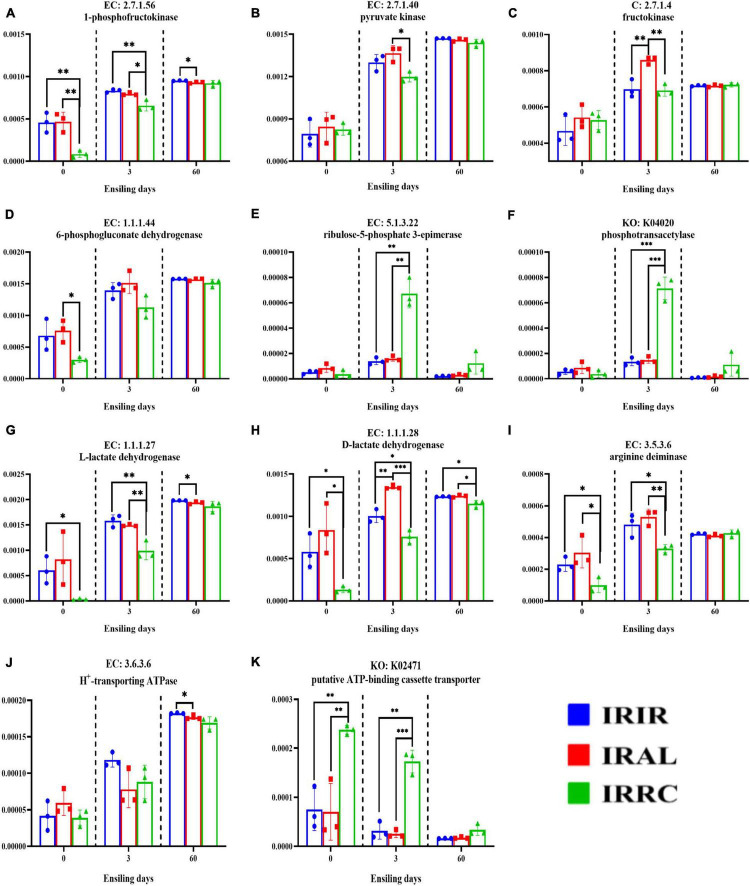
Changes of relative abundances of key enzymes **(A–K)** involved in some metabolic pathways in fresh materials and Italian ryegrass silages. EC, reference metabolic pathway highlighting numbers; KO, KEGG Orthology; KEGG, Kyoto Encyclopedia of Genes and Genomes; IRFM, fresh material of Italian ryegrass; ALFM, fresh material of alfalfa; RCFM, fresh material of red clover; IRIR, sterile Italian ryegrass inoculated by epiphytic bacteria from Italian ryegrass; IRAL, sterile Italian ryegrass inoculated by epiphytic bacteria from alfalfa; IRRC, sterile Italian ryegrass inoculated by epiphytic bacteria from red clover; 3, 3 days of ensiling; 60, 60 days of ensiling. *, 0.01 < *p* < 0.05; ^**^, 0.001 < *p* < 0.01; ^***^, *p* < 0.001.

As seen in Figure 6, the IRAL group had significantly (*p* < 0.05) higher abundances of fructokinase, 1-phosphofructokinase, pyruvate kinase, L-lactate dehydrogenase, and D-lactate dehydrogenase than the IRRC group on day 3. The highest (*p* < 0.01) abundances of fructokinase and D-lactate dehydrogenase were found in the IRAL-3 group. The IRRC group had the highest (*p* < 0.01) abundances of ribulose-5-phosphate 3-epimerase, phosphotransacetylase, and putative ATP-binding cassette transporter (ABC transporter) after 3 days. After 3 days of ensiling, a significantly (*p* < 0.05) higher abundance of arginine deiminase (ADI) was found in the IRIR and IRAL groups compared with the IRRC group.

## Discussion

Ensiling is an anaerobic fermentation process in which microbes are involved and play important roles in deciding the silage quality by interacting with chemical compositions contained in the raw materials. To investigate the contribution of epiphytic microbiota from different legume forages to the fermentative products in Italian ryegrass silage, the dynamic chemical compositions, bacterial communities, and metabolic pathways during Italian ryegrass ensiling were evaluated.

### Chemical Components and Microbial Populations of Fresh Forages and Sterile Italian Ryegrass

Ensiling is a LAB-driven fermentation process, and the minimum requirement for LAB population on fresh material should be higher than 5.00 log_10_ cfu/g FW (Wang et al., 2017). Hence, the lower LAB count (<5.00 log_10_ cfu/g FW) in red clover than alfalfa and Italian ryegrass may indicate that the epiphytic microbiota on red clover was not conducive to promoting the lactic acid fermentation during ensiling. Besides, higher populations (>6.40 log_10_ cfu/g FW) of undesirable microorganisms including aerobic bacteria, yeasts, and Enterobacteriaceae in three fresh materials may be adverse to the lactic acid fermentation after ensiling, because they would compete with LAB for the limited WSC contents.

The optimal WSC content of raw material should be higher than 50 g/kg DM (Li et al., 2019). Herein, the WSC content of fresh Italian ryegrass satisfied that requirement. This indicated that Italian ryegrass is suitable for assessing the fermentative products and bacterial community dynamics in response to exogenous microbiota because sufficient fermentation substrate can be provided for microorganisms during the ensiling.

The similar chemical compositions between sterile and fresh Italian ryegrass suggested that the sterilized condition that we used would not significantly change the chemical compositions of raw material. This is the prerequisite for evaluating the single contribution of exogenous microbiota to fermentation quality on the same raw material. Similarly, Wang et al. (2020a) reported that the γ-ray irradiation condition (32 kGy for 4 h) would not obviously alter the chemical compositions and enzyme properties of the substrate, and the γ-ray irradiation method was more efficient than heating and chemical additives. Furthermore, the undetectable microbes in sterile Italian ryegrass indicated that the method of γ-ray irradiation sterilization could guarantee that the epiphytic microbes of raw material are inactivated after irradiation.

### The Alpha Diversities of Fresh Materials and Silages

In the current study, with the number of reads sampled increasing, the rarefaction curves in all samples exhibited an upward trend during the initial stage of detection and then remained stable at the end. This indicated that the amount of sequencing data produced was reasonable and can fully reflect the diversity of the bacterial community in each sample. More sequencing data (>30,000) would only generate a few species (OTU) that would not influence the overall evaluation of bacterial community structure in samples. Furthermore, the Shannon curves reached stable levels at the early stage of detection. This also proved that the sequencing data obtained could provide the majority of information about the bacterial communities in samples.

In this study, fresh materials had similar alpha diversities with silage samples on day 3 (except the IRRC-3 group) but higher alpha diversities (mainly include OTUs, Sobs, Shannon, Ace, Chao1, and Shannoneven) than silage samples on day 60. It was probably because the fresh materials existed in the aerobic and neutral environment that benefited the flourishment of epiphytic aerobic microorganisms. At the beginning of fermentation, the internal environment in silage samples has not yet become the strictly anaerobic state. However, the acidic and anaerobic environments in silage samples were basically formed after 60 days of ensiling, leading to a remarkable decrease in the bacterial diversity. This also indicated that the acidic and anaerobic environmental stresses had a great impact on the reproduction and growth of microorganisms in silages, and this effect was more obvious at the late stage of fermentation. In particular, the IRRC group had lower alpha diversity than the IRIR and IRAL groups on day 3, which was evidenced by the highest Simpson and lowest Shannon and Shannoneven indices. According to the results in bacterial compositions on the genus level, it may be because the undesirable genus *Hafnia-Obesumbacterium* in IRRC-3 accounted for a large proportion in the bacterial community, resulting in a relatively homogenous community of bacteria. This was different from the results of Du et al. (2021), who found that the lowest alpha diversity was observed in the quality silage due to the large proportions of beneficial microorganisms. Hence, the alpha diversity indices cannot be directly used as a judgment to assess the silage quality, and more attention should be paid to the specific bacterial community compositions and their metabolites. Moreover, the higher Coverage values for all samples suggested that the sampling depth had adequately captured most of the bacterial communities and was sufficient for reliable analysis of the bacterial community.

### The Bacterial Community Compositions in the Fresh Materials and Silages

In the current study, Proteobacteria and Firmicutes were both the predominant phyla in the IRFM and ALFM groups. Proteobacteria are critical in degrading organic matter and accelerating nitrogen and carbon cycles (Ma et al., 2018). Regarding Firmicutes, their acid hydrolytic function plays a vital role in various anaerobic environments, and multifarious enzymes could be produced by Firmicutes, such as proteases, cellulases, and other extracellular enzymes (Wang et al., 2020b). Unlike the IRFM and ALFM groups, Proteobacteria was the only dominant phylum in the RCFM group. This difference might be attributed to the chemical compositions of different fresh materials and environmental factors (Wang et al., 2020a). After the ensiling process, the relative abundances of Firmicutes phylum in the treated groups on days 3 and 60 were increased. It was probably because the anaerobic and acidic environments during the fermentation were conducive to the growth and reproduction of Firmicutes (Keshri et al., 2018).

Larger proportions of *Pseudomonas* were found in all fresh materials. *Pseudomonas* can survive in the anaerobic environment, and their presence in silages was detrimental due to the generation of biogenic amines resulting in the decline of CP (Duniere et al., 2013). The predominant genus in RCFM was *Methylobacterium*, which are aerobic bacteria and strictly neutrophilic, and has been reported in various forage silages (Ogunade et al., 2018; Wang et al., 2019). Furthermore, a higher proportion of *Sphingomonas* was found in RCFM. *Sphingomonas* are aerobic, Gram-negative, and pathogenic bacteria, and they could metabolize extensive xenobiotic compounds (Wang et al., 2022). Noteworthy, *Sphingomonas* and *Methylobacterium* are both harmful bacteria, which can thrive well in humid and cool environments (Guan et al., 2018).

After the ensiling process, the relative abundances of *Lactobacillus* in the IRIR and IRAL groups were higher than those in the IRRC group on days 3 and 60. *Lactobacillus* could utilize one molecule of glucose to produce two molecules of lactic acid. Some aerobic microorganisms and plant cells consume oxygen during the initial stage of fermentation, and then species of *Lactobacillus* rapidly grow and reproduction, while generating a large amount of lactic acid to reduce pH values of silage. At last, the pathogenic microorganisms (e.g., *Clostridium*) are restricted (Duniere et al., 2013). The relative abundance of *Hafnia-Obesumbacterium* in the IRRC group was higher than that in the IRIR and IRAL groups during the fermentation. Limited researches had detected the existence of *Hafnia-Obesumbacterium* in silages. Wang et al. (2020b) described that *Hafnia-Obesumbacterium* belongs to enterobacteria, and they could accelerate the proteolytic activity during ensiling. However, inhibiting the population of *Hafnia-Obesumbacterium* seems to be a difficult task. Zhao et al. (2021) found that the commercial LAB inoculants notably enhanced the production of lactic acid and reduced pH values but cannot suppress the flourishment of *Hafnia-Obesumbacterium*. Hence, the research about controlling the *Hafnia-Obesumbacterium* population in silages needs to be further conducted.

There was no difference in the relative abundances of *Lactococcus* among the three treated groups on day 3, although IRFM and ALFM had higher abundances of *Lactococcus* than RCFM. This indicated that the abundance of *Lactococcus* was more correlated with the chemical compositions in the substrate during ensiling. It was speculated that the sufficient WSC content in substrates could greatly promote the growth of *Lactococcus* during ensiling, even if their populations in raw materials were very low. The relative abundances of Enterobacteriaceae in the IRIR and IRAL groups were higher than those in the IRRC group on day 3. Generally, Enterobacteriaceae are regarded as undesirable bacteria, because they could thrive in anaerobic and weak acidic environments, compete with LAB for fermentable substrates, and convert lactic acid and WSC into acetic acid, succinic acids, ethanol, or 2,3-butanediol, leading to nutritional loss (Sun et al., 2021). The relative abundance of *Weissella* in the IRAL group was higher than that in the IRIR and IRRC groups on day 3. *Weissella* is reported as the initial colonizer microorganisms during the ensiling and is obligate hetero-fermentative bacteria that mainly metabolize WSC to acetate and lactate, and the acidic condition is adverse to their growth (Graf et al., 2016).

The silage quality can be reflected through the dynamic changes of bacterial communities during ensiling. According to the results of the community heatmap and hierarchical cluster analysis, the bacterial community in the RCFM group was different from that in the IRFM and ALFM groups. It might be because genera *Methylobacterium*, *Sphingomonas*, *Novosphingobium*, and *Rhizobium* were predominant and unique in the RCFM group.

All the silage samples were well separated from the raw materials and divided into different quadrants in the PCoA plot, indicating that ensiling time and exogenous microbiota had huge impacts on the bacterial community structure. Besides, the similar epiphytic bacteria on the IRFM and ALFM groups may result in a similar impact on the silage quality of Italian ryegrass, because the IRIR and IRAL groups were close together on the same ensiling day in the PCoA plot, which can be attributed to the higher abundances of *Lactobacillus* in the IRIR and IRAL groups.

### Fermentation Characteristics and Their Correlations With Bacterial Community Compositions

The STIR group remained in an unfermented state and had similar chemical compositions with fresh Italian ryegrass during the entire ensiling process. This similarity indicated that γ-ray irradiation with optimal dose could successfully separate the microbial and chemical compositions of herbage.

During the initial 3 days of fermentation, lactic acid contents accumulated rapidly, and pH values declined in the three treated groups. It was because the forages are chopped into small pieces to ensure the rapid release of plant juice, which promotes the growth, reproduction, and metabolism of LAB at the early stage. In the IRAL and IRRC groups, the reduction in lactic acid concentrations on day 30 of ensiling might be caused by the reduced population and activity of LAB and dominance of yeasts, enterobacteria, and clostridia evidenced by the production of acetic acid, ethanol, and NH_3_–N from the silages. During the initial stage, the IRAL group had higher lactic acid contents than the IRIR and IRRC groups. It might be related to the higher abundance of *Weissella* that existed in the IRAL-3 group. Cai et al. (1998) reported that species of *Weissella* could stimulate lactic acid fermentation at the early stage of ensiling process. After 60 days of fermentation, the IRIR and IRAL groups had higher lactic acid contents and lower pH values than the IRRC group. This may be due to higher abundances of *Lactobacillus* existing in the IRIR and IRAL groups, while the undesirable genus *Hafnia-Obesumbacterium* was predominant in the IRRC group on day 60.

During the entire ensiling process, the contents of acetic acid in the three treated groups gradually increased. There are three possible reasons for this phenomenon. Firstly, the lactic acid may be converted by undesirable microorganisms into acetic acid. Similarly, Parvin and Nishino (2009) observed that lactic acid was metabolized by acetic acid-producing bacteria to acetic acid when sugar was insufficient. Thus, higher acetic acid contents in the IRRC-60 group may be attributed to the predominant genus *Hafnia-Obesumbacterium*, which belong to enterobacteria and can metabolize lactic acid and WSC to acetic acid or other products during fermentation (Wang et al., 2020b; Sun et al., 2021). Secondly, the metabolic products of some species in *Lactobacillus* may be changed. Li and Nishino (2013) reported that the accumulation of acetic acid was probably owing to the increase of *Lactobacillus plantarum*, which is regarded as a facultatively heterofermentative species and could produce acetic acid during ensiling. This was also in agreement with the results that the IRIR and IRAL groups had much higher abundances of *Lactobacillus* on day 60. Lastly, the high moisture content (>75%) in fresh Italian ryegrass was conducive to the production of acetic acid during ensiling (Li and Nishino, 2013). Notably, the acetic acid contents in the IRIR and IRAL groups were lower than those in the IRRC group. It was probably because the activity and populations of acetic acid-producing bacteria in the IRIR and IRAL groups were inhibited by the lower-pH acidic environment. This was also consistent with the lower Enterobacteriaceae populations in the IRIR and IRAL groups on days 3 and 60.

Greater losses of DM and energy in silages can result from the high production of ethanol. Kung et al. (2018) found that over 30 ∼ 40 g/kg DM of ethanol content might be linked with the action of yeasts. In the current study, ethanol contents in all treated silages were lower than 23 g/kg DM, suggesting that ethanol was mainly generated by other microorganisms (e.g., hetero-fermentative LAB, and enterobacteria). IRRC had higher ethanol concentrations than IRAL and IRIR on day 60. This may be because the higher proportions of *Hafnia-Obesumbacterium* existed in the IRRC group.

The DM contents in the STIR group remained at a stable level as compared with the fresh Italian ryegrass, which indirectly demonstrated that appropriate γ-ray irradiation could successfully inactivate the microorganisms in forages, thus preventing the fermentation process. The DM contents of all fermented groups were reduced after ensiling, which is indicative of the microbial breakdown of nutrients into carbon dioxide and water. On day 3 or 60, the higher DM contents in IRAL than the IRIR and IRRC groups could be ascribed to the rapid decline of pH values at the early stage in the IRAL group, inhibiting the growth of undesirable microorganisms and preserving more nutrients in silages.

Ammonia nitrogen in silage reflects the level of protein degradation that can reduce the nutritional value of forage. All the fermented silages had lower NH_3_–N concentrations; less than 100 g/kg TN is considered by McDonald et al. (2002) to be acceptable for good fermentation in silages. Compared with the IRIR and IRRC groups on day 3 or 60, the lower NH_3_–N concentrations in IRAL were mainly correlated with the rapid accumulation of lactic acid and quick decline of pH values during the initial stage of ensiling, which suppressed the enzymatic activity in plant and microorganisms.

Greater WSC concentration of silages may be more nutritionally favorable for ruminants (Wang et al., 2022). The WSC levels in the STIR group remained steady during ensiling. This indirectly proved that the STIR group was not fermented. Besides, IRAL had a higher WSC concentration than IRRC and IRIR after 3 days of ensiling. This might be because the rapid acidification restricted the substrate consumption of aerobic microorganisms at the beginning of fermentation, thus saving more WSC contents in silages.

Beneficial microorganisms could contribute to the improvement of fermentation quality by producing a variety of desirable metabolites. In the current study, IRIR and IRAL had higher LAB counts than IRRC. It was in accordance with the results that the IRIR and IRAL groups had a much higher abundance of *Lactobacillus* than the IRRC group. More importantly, the highest LAB counts in the IRAL-3 group could partly explain the rapid production of lactic acid in the IRAL-3 group. Fast initial acidification is critical to control the populations of undesirable microorganisms. The higher Enterobacteriaceae and yeast populations in the IRRC group indirectly indicated that inoculating the epiphytic microbiota from red clover cannot promote the rapid accumulation of lactic acid in Italian ryegrass silages. This was correlated with the amount, species, and activity of exogenous microbiota.

After 3 days of ensiling, the negative correlation between the abundance of *Weissella* and pH values demonstrated that species of *Weissella* played an important role in reducing pH values at the early stage. *Hafnia-Obesumbacterium* had a positive correlation with acetic acid, NH_3_–N, and ethanol contents on day 60. This proved that the presence of *Hafnia-Obesumbacterium* during ensiling was adverse to silage fermentation. The negative relationship between the abundance of *Lactobacillus* and pH values on day 60 indicated that *Lactobacillus* was critical in producing lactic acid at the end of fermentation due to their acid resistance.

The arrow direction of *Hafnia-Obesumbacterium* showed an acute angle correlation with arrow directions of ethanol and pH values, indicating that the existence of *Hafnia-Obesumbacterium* contributed to the production of ethanol and impaired the acidification during fermentation. Moreover, the obtuse angle relationship between *Hafnia-Obesumbacterium* and DM arrow directions, and the acute angle relationship between *Hafnia-Obesumbacterium* and NH_3_–N arrow directions proved that the presence of *Hafnia-Obesumbacterium* may accelerate the DM loss and protein degradation during ensiling.

### Kyoto Encyclopedia of Genes and Genomes Metabolic Pathways of Bacterial Community in the Raw Materials and Silages

KEGG is a bioinformatics resource for understanding the functions and utilities of cells and organisms from both high-level and genomic perspectives. Predicting the functional shifts of the bacterial community is conducive to assess the influence of microbes on the dynamic changes of silage quality. Thus, KEGG analysis based on Tax4Fun was applied to evaluate the effects of exogenous microbiota on metabolic characteristics in Italian ryegrass silage.

Before the ensiling process, the different pathway levels in three fresh materials were mainly correlated with the diversity and richness of the epiphytic bacterial community. After 3 days of ensiling, the IRIR and IRAL groups had lower abundances of “Cellular Process” and “Environmental Information Processing” and higher abundances of “Genetic Information Processing” and “Metabolism” than the IRRC group. Based on the fermentation characteristics in different treatments, the abovementioned results indicated that the exogenous microbiota from Italian ryegrass and alfalfa improved the fermentation quality of Italian ryegrass primarily through altering the cell characteristics, inhibiting the membrane transport and signal transduction of undesirable bacteria, and accelerating the proliferation rate and metabolism level of beneficial bacteria like LAB strains. After 60 days of ensiling, a low variation of metabolic pathways on the first level is found among the different treatments. It may be because the stable internal environment was already formed at the final stage of fermentation.

The carbohydrate metabolism mainly contained gluconeogenesis and glycolysis metabolism (Kanehisa and Goto, 2000). In this study, the “Carbohydrate metabolism” pathways in different treatments were promoted after ensiling. This indicated that the microorganisms in Italian ryegrass silages, containing *Lactobacillus* and *Hafnia-Obesumbacterium*, had a higher capacity to metabolize WSC than the epiphytic bacteria on fresh materials. They competed with epiphytic microorganisms to consume fermentable substrates during the ensiling. After 3 days of fermentation, IRAL had higher abundances of “Nucleotide metabolism” and “Replication and repair” than the IRRC group. Kilstrup et al. (2005) reported that nucleotides could be utilized to synthesize and replicate RNA and DNA and provide the main energy for cellular processes. This demonstrated that LAB strains in the IRAL-3 group multiplied rapidly during the early stage of fermentation, which was in accordance with the highest LAB populations in the IRAL-3 group. Furthermore, the relative abundances of “Membrane transport” and “Signal transduction” in IRAL-3 were less than those in IRRC-3. It was in agreement with the reports of Keshri et al. (2019), who found that a higher abundance of transporters was observed in untreated silage, and it might be related to a symbiotic relationship in the bacterial community. In fact, the heterogeneous aerobic and facultative anaerobic microorganisms dominated the microbial community at the early stage of ensiling, which produced carbon dioxide and decreased the O_2_ concentrations trapped in the silo. During this period, the metabolic products generated by some microbes might be utilized by other undesirable microorganisms through transporters to acquire essential nutrients for growth and energy. With the pH value declining, the bacterial community in silage becomes more homogeneous and mainly occupied by LAB. As a result, fewer transporters are needed. In the IRAL group, the pH value was sharply reduced on day 1, and *Lactobacillus* and *Weissella* accounted for the majority of the whole bacterial community, so less transport function was needed at the early stage of fermentation. The higher abundance of “Signal transduction” in the IRRC group than the IRIR and IRAL groups on day 3 also proved that the membrane, intracellular structural molecules, and ion channel pores in the IRRC group were markedly different from those in the IRIR and IRAL groups during the initial stage of fermentation.

The carbohydrate metabolic pathways were specifically analyzed on the third pathway level. Although the carbohydrate metabolism on the second pathway level basically remained stable, the higher variation of specific carbohydrate metabolism on the third level was observed on day 3. This indicated that the carbohydrate metabolic pathway of the bacterial community in silage indeed varied dramatically especially at the beginning of fermentation. The relative abundances of the TCA cycle in all treatments were inhibited after ensiling compared with fresh materials. It might be associated with the consumption of oxygen because the TCA cycle must be carried out under aerobic conditions. Without oxygen, the removed hydrogen ions cannot enter the respiratory chain for complete oxidation (Banfalvi, 2010). On day 3, the lower abundance of TCA pathway in the IRAL group than the IRRC group indicated that the IRAL group produced larger amounts of lactic acid and established the anaerobic environment more rapidly at the beginning of ensiling. Besides, the IRRC group had higher abundances of pentose and glucuronate interconversions, glyoxylate and dicarboxylate metabolism, and C5-branched dibasic acid metabolism than the IRAL group after 3 days of fermentation. It was suggested that these substrates were more conducive to the undesirable bacteria. In the current study, there was no significant difference in starch and sucrose metabolism among the treatments on day 3, indicating that starch and sucrose were not the first substrates for the growth and proliferation of LAB strains particularly at the initial stage of ensiling. This was consistent with the reports of Shao et al. (2003), who found that glucose was the first fermentation substrate and was more susceptible to degradation relative to other carbohydrate fractions during fermentation.

Some metabolic pathways with notable differences on the second level were further analyzed on the third pathway level. These metabolic pathways were mainly involved in nucleotide metabolism (purine metabolism and pyrimidine metabolism), membrane transport (ABC transporters and bacterial secretion system), replication and repair (DNA replication, nucleotide excision repair, mismatch repair, and homologous recombination), and signal transduction (two-component system). Their relative abundances varied considerably during the initial stage but basically remained stable level at the final stage of ensiling. This indicated that more attention should be paid to modulating the early stage of ensiling because this stage included a series of biochemical reactions and changes and was of great importance to the final quality of silage. Herein, higher abundances of purine metabolism and pyrimidine metabolism in the IRAL-3 group than in the IRRC-3 group could be explained by the rapid growth and proliferation of LAB strains in the IRAL-3 group. After the ensiling process, the relative abundances of ABC transporters in all treatments were promoted, while the relative abundances of the bacterial secretion system in all treatments were inhibited, especially on day 60. This indicated that the membrane transport of the bacterial community in silages was mainly via ABC transporters rather than the bacterial secretion system, and ABC transporters were more suitable to the silage environment. Moreover, IRAL-3 had fewer ABC transporters and a bacterial secretion system than IRRC-3. This was consistent with the abovementioned result that the IRAL-3 group required less transport function than the IRRC-3 group during the ensiling due to the rapid acidification and large amounts of LAB that existed at the early stage in the IRAL-3 group. Also, the IRAL-3 group had higher abundances of DNA replication, nucleotide excision repair, mismatch repair, and homologous recombination than the IRRC-3 group, suggesting that the biochemical reactions involved in DNA replication and repair were mainly reflected through the rapid growth and proliferation of LAB strains (e.g., *Lactobacillus* and *Weissella*) in the IRAL-3 group. The lower abundance of the two-component system in the IRAL-3 group than the IRRC-3 group proved that the signal transduction in the IRAL-3 group was indeed inhibited, and the rapid acidification could suppress the two-component system in undesirable microorganisms.

### The Activity of Key Enzymes of Bacterial Community in the Raw Materials and Silages

The key enzymes of the bacterial community played important roles in the formation of fermentation products. It is well known that 1-phosphofructokinase, pyruvate kinase, and fructokinase are the most important enzymes in the Embden–Meyerhof pathway (EMP). Higher abundances of fructokinase, 1-phosphofructokinase, and pyruvate kinase in the IRAL-3 group than the IRRC-3 group indicated that these enzymes are critical to stimulate lactic acid fermentation during the early stage of ensiling. Notably, the highest abundance of fructokinase in the IRAL-3 group may result in the largest production of lactic acid in the IRAL-3 group. The heterofermentative LAB that produces acetic acid possesses the pentose phosphate pathway (PPP) (Abdel-Rahman et al., 2010). The 6-phosphogluconate dehydrogenase, ribulose-5-phosphate 3-epimerase, and phosphotransacetylase were mainly involved in the PPP pathway and heterofermentative process. Thus, the highest abundances of ribulose-5-phosphate 3-epimerase and phosphotransacetylase in the IRRC-3 group may lead to the massive acetic acid concentrations in the IRRC group at the final stage. The higher abundances of L-lactate dehydrogenase and D-lactate dehydrogenase in the IRAL-3 group than the IRRC-3 group were in accordance with the massive production of lactic acid in the IRAL group during the early stage. The highest abundance of D-lactate dehydrogenase in the IRAL-3 group indicated that D-lactate dehydrogenase played a more important role in producing lactic acid at the beginning of ensiling. Notably, lactic acid is metabolized as the end product of glycolysis in LAB. This indicates that LAB during growth confronts an environment that continuously increases in acidity. Actually, acid stress is a kind of self-imposed force for LAB due to its metabolism properties and function. The extreme acid condition could disturb the metabolism of LAB or even kill them. Hence, it is reasonable to assume that low pH can induce other systems in LAB to buffer lactic acid acidity, such as ADI pathways. In the ADI pathway, arginine is converted to ornithine and subsequently results in the production of ATP, CO_2_, and NH_3_ (Liu et al., 2014). After 3 days of ensiling, a higher abundance of ADI was found in the IRIR and IRAL groups compared with the IRRC group. This conformed to our findings that IRIR and IRAL had higher populations of LAB than IRRC on day 3, which was probably because the LAB strains in the IRIR and IRAL groups were more resistant to acid. The H^+^-transporting ATPase and putative ATP-binding cassette transporter are both the key enzymes in membrane transport systems (Konings, 2002). However, a higher variation among treatments was only observed in the relative abundance of putative ATP-binding cassette transporter rather than H^+^-transporting ATPase. A higher abundance of ABC transporter in the IRRC-3 group proved the abovementioned results that the membrane transport of undesirable bacteria (e.g., *Hafnia-Obesumbacterium*) in the IRRC-3 group was stronger at the beginning of fermentation.

## Conclusion

Inoculating the epiphytic microbiota from different legume forages dramatically changed the fermentative products and bacterial community compositions and predicted metabolic pathways in Italian ryegrass silage. IRAL had notably higher lactic acid contents than IRIR and IRRC on day 3. IRRC had the lowest lactic acid contents and the highest pH values, acetic acid, ethanol and ammonia nitrogen contents, and Enterobacteriaceae populations on day 60. On days 3 and 60, *Lactobacillus* was the dominant genus in IRIR and IRAL, while *Hafnia-Obesumbacterium* was predominant in IRRC. Moreover, IRIR and IRAL had significantly higher abundances of “Metabolism” and lower abundances of “Membrane transport” and “ABC transporters” than IRRC on day 3. IRIR and IRAL had evidently lower abundances of ribulose-5-phosphate 3-epimerase, phosphotransacetylase, and putative ATP-binding cassette transporter and a higher abundance of ADI than IRRC on day 3. IRAL had the highest abundance of fructokinase on day 3. The microbial factors that result in the differences in fermentative products between legume forages and grass were revealed. Knowledge regarding the effect of epiphytic microbiota could provide more insights into the improvement of silage quality.

## Data Availability Statement

The datasets presented in this study can be found in online repositories. The names of the repository/repositories and accession number(s) can be found in the article/supplementary material.

## Author Contributions

SW and TS were responsible for the study design. SW, JL, ZD, and JZ carried out all the experiments. SW analyzed the data and wrote the manuscript. JL, ZD, and JZ revised the manuscript. All authors read and approved the final manuscript.

## Conflict of Interest

The authors declare that the research was conducted in the absence of any commercial or financial relationships that could be construed as a potential conflict of interest.

## Publisher’s Note

All claims expressed in this article are solely those of the authors and do not necessarily represent those of their affiliated organizations, or those of the publisher, the editors and the reviewers. Any product that may be evaluated in this article, or claim that may be made by its manufacturer, is not guaranteed or endorsed by the publisher.
